# Suboptimal Consumption of Relevant Immune System Micronutrients Is Associated with a Worse Impact of COVID-19 in Spanish Populations

**DOI:** 10.3390/nu14112254

**Published:** 2022-05-27

**Authors:** Sebastià Galmés, Andreu Palou, Francisca Serra

**Affiliations:** 1 Laboratory of Molecular Biology, Nutrition and Biotechnology (Nutrigenomics, Biomarkers and Risk Evaluation–NuBE), University of the Balearic Islands, 07122 Palma, Spain; andreu.palou@uib.es (A.P.); francisca.serra@uib.es (F.S.); 2 Centro de Investigación Biomédica en Red de Fisiopatología de la Obesidad y Nutrición, Instituto de Salud Carlos III, 28029 Madrid, Spain; 3 Health Research Institute of the Balearic Islands (IdISBa), 07120 Palma, Spain; 4 Alimentómica S.L., Spin-off n.1 of the University of the Balearic Islands, 07121 Palma, Spain

**Keywords:** nutrition, micronutrients, vitamin D, COVID-19, public health, epidemiology

## Abstract

Coronavirus disease 2019 (COVID-19) has caused a global health crisis and the factors behind its differential impact on COVID-19 among populations are still being known. Geographical differences in nutrient profile could be a relevant factor, especially considering that scientific evidence supports that 10 micronutrients are essential for proper immune system function. This study aims to evaluate these micronutrient intakes in the territories of Spain and to analyze their relationship with epidemiological indicators of COVID-19 from the first two waves of COVID-19, when neither specific vaccines nor drugs had yet come into play. Results showed that vitamin D, A, B_9_, and zinc intakes were particularly insufficient in Spain. The joint intake of these four micronutrients was lower in regions with the highest COVID-19 incidence and mortality, and of particular importance, was the insufficient intake of vitamin D. A pattern of food consumption associated with lower COVID-19 impact was observed. In conclusion, the results show the relevance of the optimal consumption of foods rich in essential nutrients for the immune system. Therefore, this assessment could serve to launch specific dietary recommendations to strengthen the immune system in Spanish territories to better face potential new COVID-19 variants and/or further infectious diseases.

## 1. Introduction

Since the first cases of coronavirus disease (COVID-19) were detected in December 2019, caused by the newly identified coronavirus SARS-CoV-2, the disease has spread all over the world and is still active. The COVID-19 pandemic has caused about 495 million confirmed cases and has exceeded six million official deaths around the world (see updated information in [1]) while reality (estimated from the excess mortality) more than tripled this [2]. In Spain, the expansion of the virus started in January 2020. Since then, it has spread in waves of different magnitude and duration, with more than 11 million cases and 101,416 deaths officially reported to WHO by 15 March 2022.

Always keeping in mind that some other fundamental aspects are the main contributors to avoiding COVID-19 expansion (including vaccination, antiviral medicines, political decisions on prevention, healthcare system capacity or individual behavior, among others), optimal nutritional status emerges as a public health issue of relevance to prevent and fight against infectious diseases [3]. However, the sparse data on the long-term duration of protective immunity, together with the appearance of emerging immune escape variants put pressure on the immune system. In this context, nutritional factors that determine its proper functioning could be key elements to improve prevention and minimize viral impact.

Furthermore, balanced nutrition is also crucial in the prevention of metabolic disorders such as obesity or metabolic syndrome, which indirectly can compromise the proper functioning of the immune system [4]. In this sense, the scientific Panel of the European Food Safety Authority (EFSA) endorses six vitamins (D [5,6], A [5], C [5,6], B_6_, B_9_ (folate), and B_12_ [7]) and four essential minerals (zinc [8], iron [9], copper [10,11], and selenium [12]) as being indispensable for the optimal working of the immune system. Specifically, there is comprehensive scientific evidence of the cause-and-effect relationship between daily intake of these nutrients and healthy immune system function. The relevance of the above-mentioned micronutrients has been recently assessed in a recent ecological study [13]. Data on micronutrient intake in the adult population from 10 European countries have shown that suboptimal intake of vitamins D, C, B_12_, and iron was associated with the higher COVID-19 incidence and/or mortality rate during the first European wave [13]. Of special interest was the observation that suboptimal intakes of vitamins D, A, B9 (folate) and zinc are present in Spain, which could be relevant to the greater impact of COVID-19 that it suffers compared to neighboring countries. Particularly relevant is vitamin D, whose deficient intake is common in Spain, especially in older people [13].

In this context, the present study aims to: (1) evaluate the intake of essential micronutrients for the immune system and; (2) study their potential relationship with COVID-19 indicators in the different regions of Spain (Autonomous Communities, ACs). In addition and associated with the different culinary habits and gastronomic wealth present throughout Spain, the aim is to identify those foods or dietary factors common in each Spanish region that could contribute to providing the essential micronutrients for the immune system and explain the differential impact of the disease caused by SARS-CoV-2 in the different regions of Spain.

## 2. Materials and Methods

### 2.1. Selection of Nutrients

The European Food Safety Authority (EFSA) endorses that there is sufficient scientific evidence to substantiate the cause–effect relationship between the intake of six vitamins and four minerals for optimal immune system function. Specifically, these 10 micronutrients are vitamin D [5,6], vitamin A [5], vitamin C [5,6], vitamin B_6_, vitamin B_9_ (folate), vitamin B_12_ [7], zinc [8], copper [10,11], iron [9], and selenium [12]. Consequently, these were the nutrients selected to analyze the intake in the Spanish population and to assess their potential relationship with epidemiological indicators of COVID-19. The list of nutrients, their Dietary Recommended Values (DRVs) for adults, and previously published intake data in Spain are shown in Table 1.

### 2.2. Micronutrient Intake in Spain and Spanish Regions (Autonomous Communities)

The micronutrient intake in Spain and ACs were obtained from the household Spanish report, containing pre-pandemic (2019) consumption data and, published by the Ministry of Agriculture, Fisheries and Food (*Ministerio de Agricultura, Pesca y Alimentación*) of the Spanish Government [20]. The report includes data on the consumption of ≈680 food items at the national level and by ACs. Foods whose consumption in Spain is relatively common and those that provide a significant amount of the above-mentioned micronutrients were specifically considered. The criteria for the selection of the food items were based on two premises. To evaluate the specific intake level of the 10 micronutrients (objective (1), food items considered rich in at least one vitamin or mineral were selected. Specifically, those foods whose intake of 100 g contributed at least 10% of the DRVs (Table 1) were chosen and used to calculate population intake. Then, a second food selection step was performed, including those foods whose consumption provided at least the average intake (observed at the national level) of one of the micronutrients considered (objective (2)). This second list of foods was used to compare nutritional profiles between areas of Spain as well as to evaluate its potential implication for COVID-19. Food consumption values (kg or L per capita/year) from the database [20] were converted to grams/day/person and, the specific intake of the micronutrients of interest was assessed from food composition data retrieved at FoodData Central of the United States Department of Agriculture (USDA, https://fdc.nal.usda.gov/ (accessed on December 2020)). Finally, the estimation of the intake of each micronutrient was obtained from the addition of the contributions of all the selected foods.

### 2.3. Epidemiological Indicators of COVID-19 in Spain

The epidemiological COVID-19 Spanish data was collected from the regular reports of the “Center for the Coordination of Health Alerts and Emergencies” of the Ministry of Health of the Government of Spain. Specifically, the accumulated incidence and the total deaths due to COVID-19 since the beginning of the pandemic have been obtained from the “Update No. 235 of the Coronavirus Disease (COVID-19) on 23 October 2020 [21] (www.mscbs.gob.es (accessed on December 2020)), which was around the exponential phase of the second wave. COVID-19 Incidence (I) is expressed as the number of cases per 100 k people (from the beginning up to 23 October 2020), and Mortality rate (M) is expressed as the number of dead people due to COVID-19 per 1 M people. Z-score values of both I and M were added to obtain the I + M index, a parameter here used to denote the penetrance status of COVID-19 in Spanish regions. Additionally, the “Update No. 203” [22] on 9 September 2020 (45 days earlier than Update No. 235) which corresponds to a previous peak of national infections has also been retrieved. Data from Update No. 203 has been used to estimate the incidence 45 days earlier (I_-45_) and the increase in incidence between the cusps of these two spikes (waves) of infections (Δ Incidence = I/I_-45_). ΔI has been used as a marker of the expansion of cases in Spain and its geographical regions. Prevalence (P) data have been obtained from the National Study of Sero-Epidemiology of infection by SARS-CoV-2 in Spain (ENE-COVID) carried out by the Ministry of Health, the Carlos III Health Institute, and the Health Services of all the ACs [23]. ENE-COVID is a sero-epidemiological longitudinal population study, in which samples of more than 72,000 people have been obtained in 4 sampling rounds and estimates the prevalence of SARS-CoV-2 infection in Spain and its regions by determining antibodies (IgG) against the virus. Therefore, the P data in each region are expressed as the estimate of the cumulative percentage of the population with a positive test for IgG against SARS-CoV-2 during the fourth round of sampling (before vaccination, data published on 15 December 2020). Spanish regional COVID-19 epidemiological indicators (P, I_-45_, I, M and I + M) are shown in Table 2.

### 2.4. Data Calculation, Presentation, and Statistical Analyses

The intake of each micronutrient in the respective 17 Spanish ACs has been calculated. Data at the national level have been calculated as the median of the individual values of each AC. The values obtained at the national level were compared with the micronutrient intake reported by previous national studies.

To standardize the different sets of variables in a comparable scale magnitude, the Z-score of the variables at the national level has been used. Thereby, the mean (μ) and population standard deviation (σ) of all variables (e.g., incidence, mortality rate, vitamin D intake, …) were calculated using the ACs individual values. Then, the Z-score of each one of the ACs was calculated (z = (x − μ)/σ), x being the specific value for one AC. Thus, positive Z-score values mean that the ACs value are over the national mean, while negative values indicate that ACs are underneath the country mean for this specific trait. The percentage of DRV accomplishment (% DRV) was calculated as the percentage of the intake (in Spain or in an AC) refereed to EFSA’s DRVs set for each micronutrient in adults (see Table 1). When there was a specific DRV for women and men, the mean of both values was used for this calculation.

Spearman correlation analyses have been carried out between epidemiological indicators and micronutrient intake. Spearman coefficients (Rho) and *p*-values have been obtained with SPSS v27 (SPSS, Chicago, IL, USA) and the correlation map was performed using R Software Package Corrplot of Statistical Tools for High-throughput data analysis (STHDA) [24].

Choropleth maps providing visual variation across the Spanish regions were designed using Microsoft Excel graphics (Microsoft 365, Redmond, Washington, DC, USA). The combined epidemiological I + M index was used as the reference for the impact of COVID-19 in the territories of Spain. Depending on the test performed, the I + M index was used as a continuous (for correlations) or categorized variable, dividing the territories into mathematical quartiles (Q), to compare them. Thus, the first quartile (Q1) was formed by the ACs with the lowest I + M index and the fourth quartile (Q4) by those with the highest I + M. Comparisons of the mean intake of nutrients between groups based on the I + M index have been statistically supported by the Student’s *t* test using Microsoft Excel formulas (Microsoft 365, Redmond, Washington, DC, USA).

Principal Component Analysis (PCA) was carried out with SPSS v27 (SPSS, Chicago, IL, USA) using consumption/intake (per year) normalized variables (Z-score). PCA was performed, including the food items whose contribution in at least one of the micronutrients was ≥ than the national average intake for this specific micronutrient. To obtain a simpler interpretation of the whole data, dimension reduction was set up for the extraction of two principal components (PC). The factor coefficients of these new data were obtained using the regression method and were graphically represented to obtain the dimensional factors that may reflect the dietary pattern of each Spanish region. Furthermore, the weight values in these two PCs for each nutritional item were obtained by Varimax rotation.

## 3. Results

### 3.1. Epidemiological Situation during the Exponential Phase of the Second Wave of COVID-19 in the Regions of Spain

The Data Update No. 235 of the Coronavirus Disease (COVID-19) from the Spanish “Center for the Coordination of Health Alerts and Emergencies” (*Centro de Coordinación de Alertas y Emergencias Sanitarias*) exposed that Spain had a total of infected people that exceeded a million. Specifically, a total of 1.046.132 confirmed cases of COVID-19 and 34.752 deaths had been officially reported since the pandemic onset. The accumulated data show that at this time, Spain was approximately in the exponential phase of the second wave of COVID-19 infections. Interestingly, Spanish regions—classified as ACs (the first-level of political and administrative division)—showed a wide range of variation in the COVID-19 epidemiological indicators (see Table 2). Particularly, the I + M index, a parameter that typifies the penetrance status of COVID-19 reflected the high variability existing between the different Spanish regions. Thus, low I + M were observed in both archipelagos (the Canary Islands and the Balearic Islands) together with some of the peripheral areas of the Iberian Peninsula, such as Galicia, the Principality of Asturias, the Valencian Community, and Andalusia. Then, an intermediate risk group appeared formed by Cantabria, the Region of Murcia and Extremadura, whose I + M index were below the national median (see Table 2). Catalonia and the Basque Country with positive I + M index, completed the group of ACs that could be considered with medium COVID-19 impact. Finally, the highest I + M index was observed in the Community of Madrid, La Rioja, Chartered Community of Navarre, Castilla-La Mancha, Aragon, and Castilla y León, the regions geographically located in the central area of the country. Therefore, the expansion of the pandemic in Spain could be influenced by the physical proximity of the regions, but also by the lifestyles and cultural characteristics of the Spanish territories, such as the distinctive food traditions (tapas and so on) or the nutritional profile of each region of Spain, which is analyzed below.

The ACs I + M index mostly correlated with I_-45_ and Prevalence (P) indicators, with very high correlation coefficients (Rho = 0.895; *p* < 0.001; Rho = 0.922; *p* < 0.001, respectively), suggesting that penetrance status of COVID-19 was highly dependent on baseline incidence (I_-45_) and immunological status of the population. In addition, ΔI showed that the incidence tendency could be relevant in those regions that still had medium or low COVID-19 impact, such as the Region of Murcia, Andalusia, Extremadura, or the Principality of Asturias as this indicator would provide information on the ease of spread of the virus in a given area in a specific time period (Table 2).

### 3.2. Intake of Essential Micronutrients for the Immune System at the National Level

The intake in Spain of the 10 micronutrients provided with foods whose intake of 100 g contributes at least 10% of the DRVs is shown in Table 1. Previously published data from different sources have also been collected and incorporated into the table, which may help to confirm the accuracy of 2019 data calculated from the national household consumption and expenditure survey. The results showed a suboptimal consumption, especially of three vitamins: vitamin D, vitamin A, and vitamin B9; and three minerals: zinc, iron, and copper, whose intake level did not reach 80% of the population recommendations.

Among these micronutrients, vitamin D was the nutrient with the greatest deficiency in Spain showing only an intake of 2.46 µg/day, reaching 16.2% of the DRVs for the adult population. Intake of zinc, vitamin A, and copper was also remarkably suboptimal, with medians of 4.42 mg/day (38.6% of DRVs), 277 µg/day (39.9% of DRVs), and 0.62 mg/day (43.5% of DRVs), respectively. Furthermore, below the recommendations but without falling below 50% of the DRVs, vitamin B_9_ (238 µg/day; 71.1% of DRVs), iron (7.96 mg/day; 72.8% of DRVs), vitamin B_6_ (1.40 mg/day; 83.2% of DRVs) and vitamin C (95.0 mg/day; 91.9% of DRVs) were found. In contrast, the median intake of selenium (92.3 µg/day; 131% of DRVs) and vitamin B_12_ (5.23 µg/day; 132% of DRVs) were above the recommendations (Table 1). In comparison with data consulted from other sources and different years, it is worth noting the similarity of the reported intake of these micronutrients [14,15,16,17,18] and particularly, the suboptimal consumption consistently reported of vitamin D, vitamin A, vitamin B_9_, and zinc.

### 3.3. Micronutrient Intake in the Spanish Autonomous Communities

The intake and the percentage of contribution to DRVs of the 10 micronutrients in each AC are shown in Table S1. The standardization of these data by the Z-score allowed contextualizing the nutritional status of each ACs to the national situation and to identify the regions with the lowest intake of each vitamin or mineral. In this analysis, special attention was paid to those micronutrients whose consumption below the recommendations was observed in the three data sets (Table 1). Therefore, the focus was on the intake of vitamin D, vitamin A, vitamin B_9_, and zinc.

**Figure 1:**
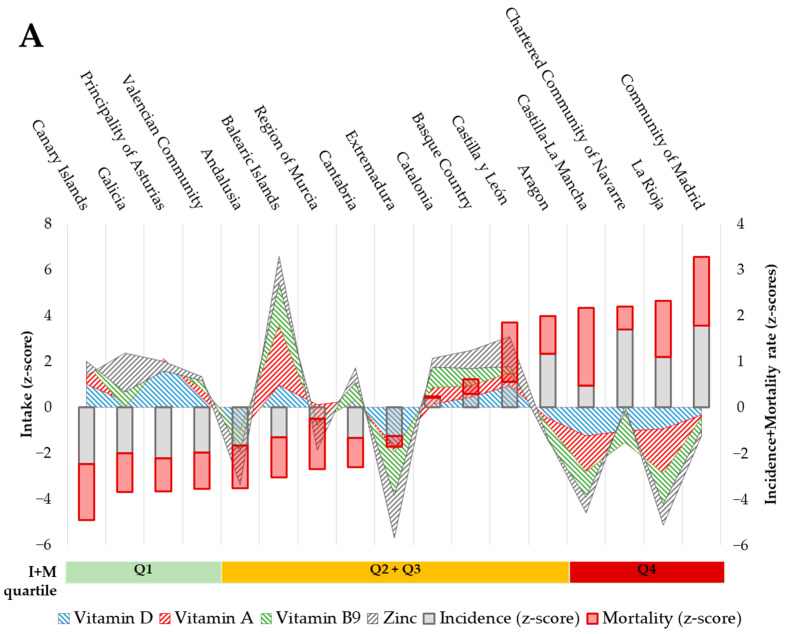

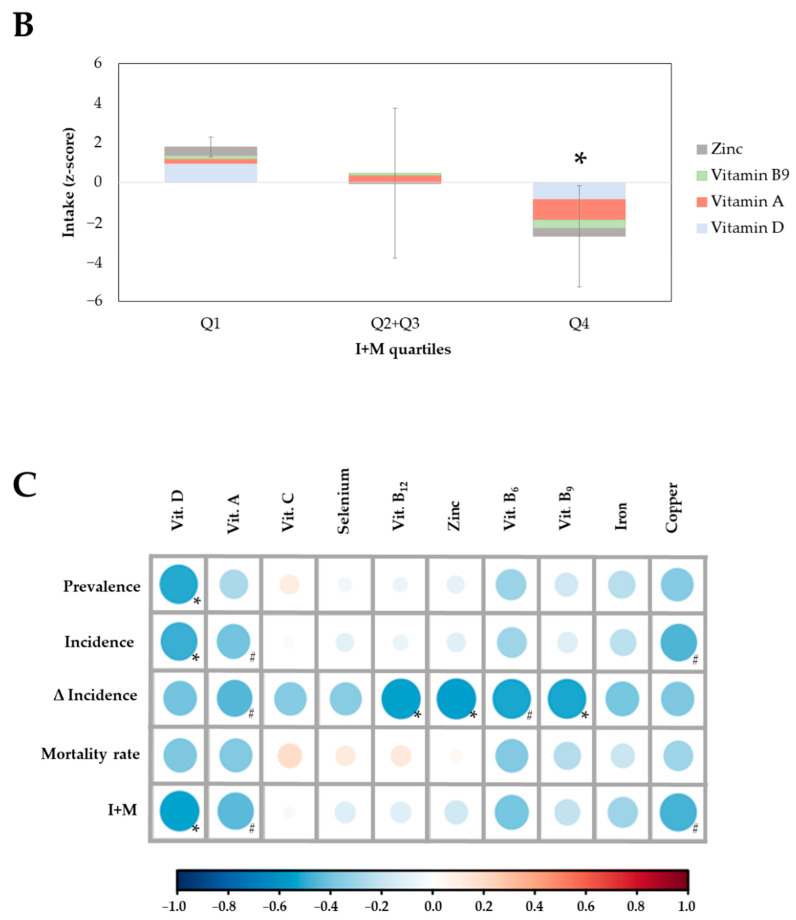
Relationship between micronutrient intake (Z-score) and the impact of COVID-19 in the Autonomous Communities. (**A**) Cumulative intake of micronutrients—vitamin D (blue shading), vitamin A (red shading), vitamin B_9_ (green shading), and zinc (grey shading)—and the impact of COVID-19 (I + M index; Incidence (gray box); mortality (red box)) in Spanish ACs. ACs appear in increasing order of I + M index. ACs data are represented as the Z-score of the Spanish intake and classified by quartiles (Q) of the I + M index. (**B**) Mean ± standard deviation of the intake of vitamin D (blue box), vitamin A (red), vitamin B_9_ (green), and zinc (grey) in the ACs in quartile 1 (Q1), quartile 2 plus 3 (Q2 + Q3) and quartile 4 (Q4). Differences among means were assessed by Student’s T test comparing Q2 + Q3 vs. Q1 and Q4 vs. Q1, and *p*-value < 0.05 are indicated by * (**C**) Spearman correlation map of the intake level (Z-score) of each micronutrient and COVID-19 Prevalence, Incidence (COVID-19 cases per 100 k population), Incidence increase (∆ Incidence, in a 1.5-month period), Mortality rate (COVID-19 deaths per 1 M population) and, Incidence and Mortality index (I + M). # Spearman correlation *p*-value < 0.1; * Spearman correlation *p*-value < 0.05.

The intake of vitamin D was relatively low in Extremadura (Z-score: −1.75), Castilla-La Mancha (−1.25), and Andalusia (−1.07) reaching approx. 15% of DRVs; while it was relatively high in the Principality of Asturias (+2.11), the Balearic Islands (+0.96), and the Canary Islands (+0.76), although this only met 17–19% of DRVs (Figure 1A). Intake of Vitamin A was relatively low in La Rioja (−1.95), Castilla-La Mancha (−1.54), and the Region of Murcia (−0.77) (covering 32–37% of DRVs), while the ACs with the highest relative intake were the Balearic and Canary Islands (+2.61 and +0.88, respectively) and Catalonia (+0.72), meeting 43–51% of DRVs. A better nutritional pattern was observed concerning the intake of vitamin B_9_; the lowest consumptions were observed in Extremadura (−1.88), La Rioja (−1.34), and Castilla-La Mancha (−1.11), which accounted for 57–63% of DRVs; in contrast, the highest intakes were reported in the Balearic Islands (+1.79), Navarre (+1.34) and Catalonia (+0.89), meeting 78–85% of DRVs. Finally, Extremadura (−1.86), Andalusia (−1.46) and the Region of Murcia (−1.11) were the regions with the lowest zinc intake (32–35% of DRVs), and Galicia (+1.71), Castilla y León (+1.30) and Balearic Islands (+1.20) were the regions of Spain with the highest intake of this mineral (43–45% of DRVs). Furthermore, regional differences could also be outlined, like Extremadura and Castilla-La Mancha, which were in the suboptimal range for more than one micronutrient, while the two archipelagos showed better nutrient profiles for more than one micronutrient (Figure 1A and Table S1). Despite the fact that the DRVs of these micronutrients were not reached in any of the regions, significant differences were observed between the cumulative intake (the sum of the Z-score values for each micronutrient, see Figure 1B) of these four micronutrients according to I + M quartiles, being significantly lower in the ACs positioned in the Q4 (value mean of −2.73) compared with the Q1 ones (1.79, *p* = 0.013).

### 3.4. Association between the Intake of Micronutrients and COVID-19 Indicators in the ACs

Potential associations between the intake of the essential micronutrients for the proper functioning of the immune system (Table 1) and epidemiological indicators of COVID-19 in the ACs (Table 2) were assessed through correlation studies. The analyses revealed inverse and significant associations between the intake of specific micronutrients and Prevalence (P), Incidence (I), Incidence Increase (∆I), and/or I + M index. Specifically, the intake of vitamin D was associated with lower P (Rho = −0.512; *p* = 0.036), I (Rho = −0.493; *p* = 0.045) and I + M (Rho = −0.534; *p* = 0.027); the intake of vitamin A was associated with lower I (Rho = −0.422; *p* = 0.092), ∆I (Rho = −0.444; *p* = 0.074) and I + M (Rho = −0.458; *p* = 0.064), although without reaching statistical significance; vitamin B_6_ (Rho = −0.468; *p* = 0.058), vitamin B_9_ (Rho = −0.510; *p* = 0.037), vitamin B_12_ (Rho = −0.522; *p* = 0.032) and zinc (Rho = −0.527; *p* = 0.030) intake levels were associated with lower ∆I. In addition, a tendency in the association between copper intake and lower I (Rho = −0.444; *p* = 0.074) and I + M (Rho = −0.456; *p* = 0.066) could be also observed (Figure 1C). Thus, ACs with a richer intake of vitamin A, vitamin B_6_, B_9_, B_12_, zinc and, especially vitamin D would show better performance in stopping the spread of the virus.

**Figure 2:**
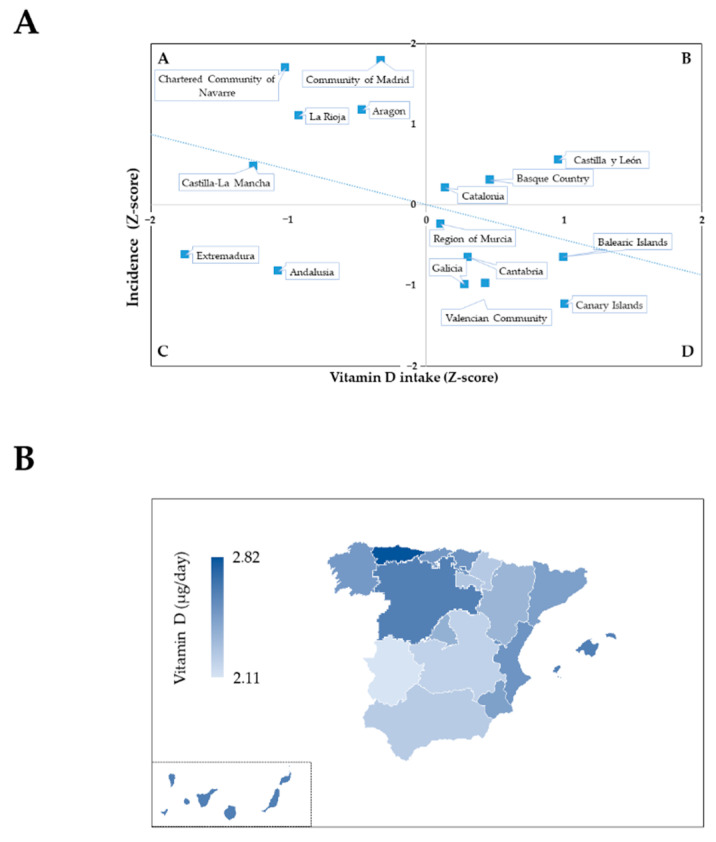
Relationship between intake of vitamin D and COVID-19 incidence in the Autonomous Communities of Spain: (**A**) Correlation between the intake of vitamin D and the Incidence of COVID-19 (both in Z-scores) in Spain; (**B**) Choropleth map of vitamin D intake (µg/day) in Spanish Autonomous Communities.

Vitamin D status seems to be a key factor in the incidence and mortality (I + M index) derived from COVID-19 (Figure 1C). Correlation studies highlighted its relevance, as a linear inverse relationship could be observed between vitamin D intake in the Spanish regions and the Incidence of COVID-19. Furthermore, the incidence was observed distributed into four quadrants according to the nutritional status of vitamin D (Figure 2A, standardized Z-score values). Those communities that have suffered the worst blow from the pandemic during the studied period appeared grouped in Quadrant A (Figure 2A), which is characterized by higher incidence (Z-score > 0) and the lowest vitamin D intake (Z-score < 0). This is in contrast to Quadrant D, which contains the ACs with the least impact of COVID-19, which were also those with the best nutritional status of vitamin D.

In addition, Quadrant B included Castilla y León, Basque Country, and Catalonia, which did not fit the correlation as they have presented high COVID-19 incidence and could be considered to have a relatively good intake of vitamin D (although they did not reach DRVs). In these ACs, other factors independent of vitamin D intake seem to be more relevant in the infection rate. Lastly, Quadrant C grouped the regions of Extremadura and Andalusia, ACs with low incidence despite suboptimal intake of vitamin D. Although, a rapid increase in infections (ΔI = 2.6, Extremadura; ΔI = 2.8, Andalusia; Table 2) has been observed in those, which would suggest that a low intake of vitamin D may contribute to COVID-19 expansion in these ACs.

Finally, the set of data was consistent with a geographical pattern, with higher vitamin D intake in maritime areas (archipelagos, Cantabrian, and Mediterranean littoral zones), which were associated with lower COVID-19 incidence (Figure 2B).

### 3.5. Analysis of Main Contributing Foods by ACs and Their Relationship with Epidemiological Indicators

**Figure 3:**
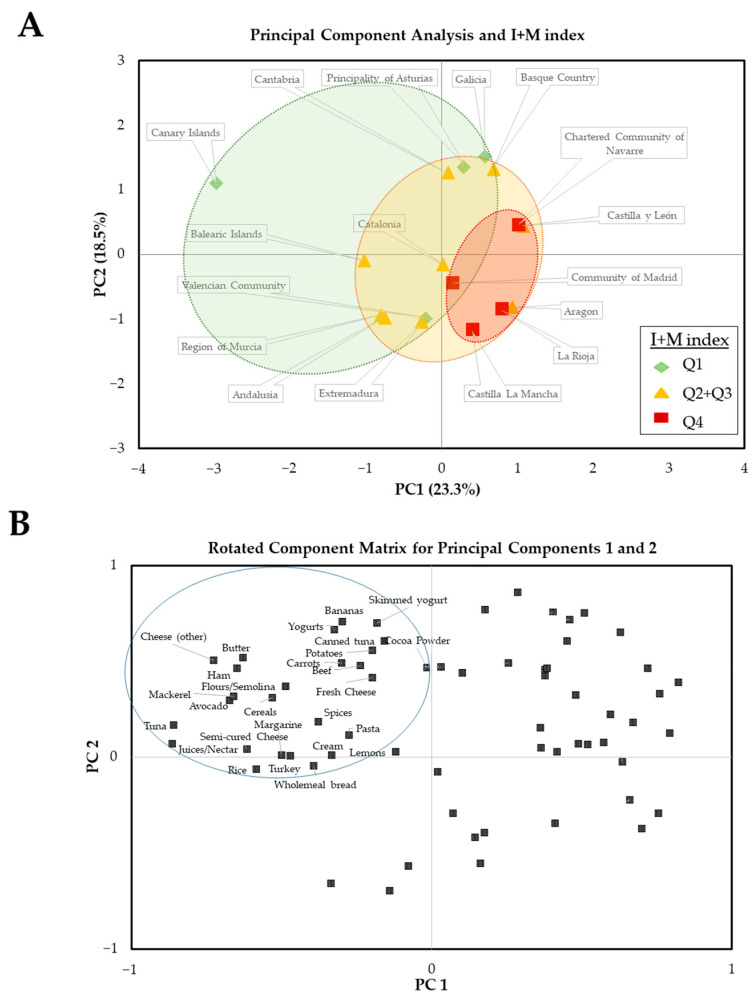
Principal Component Analysis derived matrices. (**A**) Matrix of Principal Components 1 and 2 (% of explained variability) of foods considered a source of essential micronutrients for the immune system in the Spanish regions and, classified according to the tercile of I + M index: quartile 1 (Q1, low I + M, in green), quartiles 2 + 3 (Q2 + Q3, intermediate I + M, in yellow) and quartile 4 (Q4, high I + M, in red). (**B**) Rotated Component Matrix for Principal Components 1 and 2. The points representing food items that contribute to negative PC1 and positive PC2 (parameters associated with the lower impact of COVID-19) are labeled and circled.

The next step was to identify the specific foods whose intake could contribute to the nutritional status of the micronutrients in each AC and whether the food profile could explain the differences between autonomous communities. A total of 66 food items met the established requirements. That is, their consumption contributed to an intake higher than the national average of at least one of the 10 micronutrients studied. A PCA was carried out to extract the set of foods more involved in the principal factors and to outline differential trends among ACs. The two first components, with an eigenvalue of 15.4 and 12.2, explained 41.8% of the total variance. The third and fourth components, with eigenvalues of 10.2 and 5.6, contributed to explaining the additional 15.4% and 8.4%, respectively, of total variance. Redistribution of the factor loadings by Varimax rotation was performed to further segregate food items among the four factors. In this sense, PC1 and PC2 integrate a linear combination of the intake data and their graphical distribution is shown in Figure 3A explaining 41.8% of the total variability in the intake of foods that are a source of essential micronutrients for the immune system. PC1 contributed to explaining a higher percentage of variability (23.3%) than PC2 (18.5%). Moreover, Spearman’s correlation analysis of PCs showed a strong association of PC1 with I + M Index (Rho = 0.605; *p* = 0.010), Incidence (Rho = 0.610; *p* = 0.009), Relative Mortality (Rho = 0.752; *p* < 0.001), Prevalence (Rho = 0.579; *p*= 0.015) and the percentage of deaths vs. prevalence (Rho = 0.824; *p* < 0.001). On the other hand, PC2 was not significantly correlated with any of the parameters analyzed. When the PC scores map was represented for each AC (Figure 3A), Communities identified with Q4 for the I + M index were located near the central axis, showing positive PC1 and, mostly separated from the others (Q1 and Q2 + Q3) that were aggregated on areas involving negative PC1 or positive PC2.

Therefore, those foods that make up for lower PC1 and higher PC2 scores were further analyzed. The representation of the contribution of these foods to PC1 and PC2 is shown in Figure 3B. According to obtained rotated component values, twenty-seven foods were found in the upper left corner of the graph showing the Rotated Component Matrix for PC1 and 2 (the whole food items list included in the PCA and rotated values for PC1 and PC2 are shown in Supplementary Table S2). Therefore, the upper left quadrant highlights the potential benefits of consuming enough of the foods listed there, which would be related to a better profile of the micronutrients of interest.

The analysis of the contribution to the national intake of these 27 foods showed that 11 of them were sources of selenium, 10 of zinc, nine of vitamin A, eight of iron, six of vitamin B_6_ and vitamin B_9_, five of vitamin D, vitamin C and copper and three of vitamin B_12_.

## 4. Discussion

Balanced and healthy diets should provide sufficient amounts of essential nutrients to meet individual needs for the proper functioning of the whole organism. EFSA and other international agencies have established DRV to largely cover the nutritional needs of the general population and, in some specific cases, physiological factors (such as pregnancy or lactation) are considered in addition to age and sex [25]. Recommendations can be fitted to a “population reference intake” (PRI) or “adequate intake” (AI) depending on the available information. While the PRI defines the level of intake that meets the daily needs of the majority (97.5%) of the people in the population, the AI is defined as the average level of adequate intake based on studies or scientific observations [26]. In the present study, the approximate intake of 10 essential micronutrients for the normal functioning of the immune system, based on public food consumption data, has been assessed in the Spanish population. Following these lines, results show suboptimal intake in eight of the ten micronutrients analyzed, specifically on vitamin D, A, C, B_6_, B_9_, zinc, iron, and copper (Table 1).

The methodology carried out in this study to estimate the micronutrient intake in the Spanish population was based on the combined analysis of Spanish food consumption obtained from the 2019 annual report and food composition data. This approach may be different from conventional methods, most of which were based on direct data obtained through nutritional interviews with the subjects. To check for accuracy, comparisons of the results obtained here were carried out in parallel with others from national or territorial studies. The quality of data resulted relatively similarity results being obtained from this study compared to the published data consulted (see Table 1). The most relevant finding was to identify a suboptimal nutritional status for relevant micronutrients, with the exception of the intake of vitamin B_12_ and selenium that would accomplish the DRV threshold (>100% vs. DRVs) following the methodology carried out in our study and in agreement with published data [15,17]. The intake of vitamin B_6_, which varies between 83.2 and 112%, according to the studies considered, of iron (72.8–111%), vitamin C (69.6–110%), and copper (43.5–115%) would present levels that would range between satisfying the requirements and suboptimal consumption. In contrast, the intakes of vitamin B_9_ (48.4–71.1%), zinc (38.6–81.2%), vitamin A (39.9–77.2%), and vitamin D (14.1–16.2%) were clearly framed in suboptimal levels, both in our analysis and in the studies consulted (Table 1). Especially remarkable are the lower intakes detected for vitamin A and zinc, with ratios of 0.58 and 0.57 vs. data from other published studies.

The suboptimal intake of these micronutrients, given their relevance in the functioning of the immune system, could be a contributing factor to the greater impact of COVID-19 and the epidemiological variability observed in the territories of Spain. To go further in this hypothesis, the association between micronutrient intake and COVID-19 epidemiological indicators was studied by territory in a particular pandemic period. Specifically, the period studied was framed in the exponential phase of the second wave of contagion in Spain, located temporarily after the first summer of the pandemic with the restrictions eased, even without the use of vaccines and with the predominance of the *20 A.EU1* variant [27], which caused more severe COVID-19 than later variants. In addition, at this time, the collection of epidemiological data was systematized by the authorities, which facilitates subsequent analysis.

In relation to the epidemiological data on COVID-19 in the different regions of Spain, these were mainly assessed with the I, M and the combined I + M index. Specifically, I + M index allows the joint assessment of both incidence and mortality—despite the fact that some limitations of this index must be assumed, such as the presence of factors that differentially affect incidence/mortality. Higher indicators of incidence and mortality were observed in the central areas of the peninsula, including the ACs of the Community of Madrid, La Rioja, CC of Navarre, Castilla-La Mancha, Aragon, and Castilla y León. In general, the most affected areas (I + M > 0) also had a higher prevalence of the disease, with percentages of 8.2 to 18.6 of the population having IgG antibodies against SARS-CoV-2, according to the study of Sero-prevalence ENE [23]. In contrast, the Canary Islands, Andalusia, Galicia, Asturias, Valencian Community, and the Balearic Islands showed the lowest values of the I + M index (−2.5 to −1.5) and lower prevalence (3.8–7.1%). A special mention must be reserved for the case of Andalusia and Asturias (Table 2), as despite being identified as geographical areas with low COVID-19 impact, their prevalence was the highest in the group and both showed a high rate of increase in cases (∆I), which would be associated with a forecast for an increase in the number of cases in a short period of time.

Concerning micronutrient intake, high variability throughout the regions of Spain was identified. Furthermore, associations between nutritional status and the impact indicators of COVID-19 were observed (Figure 1). Particularly, the suboptimal intake of vitamin D was strongly associated with higher Prevalence, higher Incidence, and consequently higher I + M index in the national context (Figure 1C). Although sun exposure can be considered a source of vitamin D and Spain has considerable hours of sunlight, the use of sun creams or the lack of exposure can contribute to making dietary vitamin D the main source of its bioavailability [28,29].

According to our data and in agreement with dietary references [30], vitamin D would be the micronutrient with the greatest need to reinforce its availability in Spain. Insufficient consumption of vitamin D was observed (2.46 µg/day), which would reach 16.2% of the recommendations for adults (based on the AI of the EFSA of 15 µg daily) [30] (Table 1). Even though all the regions of Spain would present a consumption below the DRVs for vitamin D, the ACs located at Q1 in the I + M index (the Canary Islands, Galicia, Asturias, and Valencian Community) showed intakes above the national mean (Z-scores> 1). On the other hand, the ACs with the highest I + M index (allocated in Q4), showed intakes below the national mean (Z-scores < 1) (Figure 1A,B). Therefore, Spanish data support the importance of an adequate intake of vitamin D, essential for the proper functioning of the immune system [5,6] and, especially in the context of COVID-19.

Recently, scientific evidence supported the benefits of optimal consumption of this vitamin, and even its supplementation, against COVID-19. Epidemiological data indicate that European countries with lower intakes of vitamin D have been associated with more severe effects of COVID-19 (higher incidence and mortality) in the first wave of infections [13]. Furthermore, the potentiating effects of vitamin D on the immune system have been reviewed by Carr and Gombart [31] and include benefits at many levels, ranging from prevention, lesser severity, and softer symptoms caused by infection or the attenuation of long-term potential sequels (for example, persistent COVID-19). In addition, low circulating levels of 25-hydroxyvitamin D are associated with increased incidence and severity of disease triggered by SARS-CoV-2 [32,33,34,35]. Moreover, plasma concentrations of 25-hydroxyvitamin D above 30 ng/mL are associated with lesser risk for cardiovascular disease and all-cause mortality rate, pointing out that vitamin D supplementation is the most efficient way to accomplish these concentrations [36]. In this context, a randomized controlled trial carried out on health workers with the aim of analyzing the reduction in the severity of COVID-19 according to the dose of vitamin D3 (cholecalciferol) supplementation for three months showed the need for high doses of supplementation [37]. Thus, only subjects receiving higher supplementation doses (5000 IU/day) showed normalization of circulating levels of 25-hydroxy vitamin D, a lower tendency to suffer from COVID-19, and a higher propensity for asymptomatic disease [37].

In relation to the intake of vitamin D from natural sources, it is worth noting the difficulty in achieving an adequate intake of vitamin D just by diet, since very few foods suitable for human consumption are actually rich sources [38]. Specifically, only the intake of 100 g of three foods, fish liver oils (250 μg/100 g), certain types of mushrooms (21.1–58.7 μg/100 g) and some kinds of fish (5–25 μg/100 g), would cover the daily needs of vitamin D [38]. However, the sporadic (or low amount) consumption of these foods in Spain, especially fish liver oil, means there are difficulties in reaching the long-term achievement of significant quantities of dietary vitamin D intake. On the other hand, there are some examples of foods with lower content than those aforementioned, but more commonly consumed, such as cheese and eggs (1.3–2.9 μg/100 g) and fortified foods (kinds of milk and dairy products of fruit juices), that could notably contribute to the intake of this vitamin [38]. In fact, less than 4% foods of the total nutritional items analyzed in our study fulfilled the theoretical requirement to be considered as a source of this vitamin. Furthermore, after vitamin D intake calculation from composition and consumption data, only 17 commonly consumed foods in Spain contributed more than 10% of the DRVs (per 100 g of food consumed). Besides, the Spanish regions with higher vitamin D intake were located in the northern and Mediterranean coastal areas (Figure 2B). Nevertheless, the step aiming to refine the food selection, including those whose consumption provided at least the average intake (observed at the national level) of one of the micronutrients, highlighted that five (fresh cheese, other cheeses, cereals, yogurt, and mackerel) of these 17 foods rich in vitamin D would contribute to the pattern of food consumption associated with lower incidence and mortality from COVID 19 (Figure 3B), which highlights the importance of their regular consumption.

The biological plausibility of the beneficial effects can be explained by the fact that suboptimal or deficient intake of vitamin D is associated with the malfunction of the immune system and imbalances in the inflammatory state. The majority of immune cells express the vitamin D receptor and need this Vitamin to regulate their cellular activity [39]. Furthermore, vitamin D exerts immunomodulatory functions on the production of pro-inflammatory cytokines via inhibition of the activity of the renin-angiotensin system (RAS) [40]. Thus, the effect of vitamin D on the maintenance of innate immunological components and physiological defense barriers is associated with a reduced risk of suffering from infections by pathogens [41]. Furthermore, the immunomodulatory effect of vitamin D is associated with better management of the “cytokine storm” that triggers the immune system in patients affected by severe COVID-19 [42], which could explain the lower severity of SARS-CoV-2 disease triggered in a population with optimal levels of 25-hydroxy vitamin D or, in agreement with our data, the lowest incidence and mortality rate observed in regions of Spain with the highest intake of products rich in vitamin D.

The other three micronutrients, of which the intake in Spain did not meet the recommendations (vitamin A, vitamin B_9_, and zinc), are also associated with potential benefits in terms of COVID-19 in the literature. Concerning vitamin A, the intake of this vitamin was associated with increased incidence and I + M index (Figure 1C). In addition, the consumption of vitamin A was higher in the Spanish regions with the lowest I + M index (Q1) compared to those that were classified at Q4 (Figure 1A,B). Likewise, the PCA data defined that nine of twenty-seven foods located in the quadrant of negative PC1 and positive PC2 (associated with lower COVID-19 incidence and mortality) were important sources of vitamin A. These included fresh and semi-cured cheeses and the rest of the cheeses (other), potatoes carrots, cream, butter, margarine and cereals (Figure 3). Vitamin A is obtained from plant sources in its precursor form (carotenoids) and from animal-based foods in a more processed form (retinoids) [43]. Poor intake of this vitamin is associated with an increase in infections [44], which can be related to impaired functionality of primary immunity barriers [45] and the cells of the innate and adaptive immune system [46,47]. In addition, depletion of retinoic acid (the active form of the vitamin) is associated with inflammatory imbalances [48]. Furthermore, decreases of around 23% of circulating vitamin A have been recently associated with more severe COVID-19 symptomatology [49]. In contrast, vitamin A supplementation is associated with better referrals for infectious diseases, such as pneumonia associated with viral infection [50,51]. The benefits derived from the intake of vitamin A against infectious diseases could be based on its ability to increase endogenous antiviral responses—for example, mediated by IFN-I—the role of vitamin A derivatives as adjuvants of drugs against SARS-CoV-2 [52,53,54] or as a result of the immunomodulatory effect that it allows. In fact, supplementation with mega-doses of vitamin A has been proposed as an affordable complementary therapy for COVID-19 disease, despite the risk of exceeding tolerable levels, particularly in pregnancy. To enhance the production of IgG_1_ and to reduce the chances of complications during the acute and critical phase of the disease, supplementation for two days with 300,000–500,000 IU (for moderate-severe cases) and, 200,000 IU (for mild cases) has been proposed [55]. Besides, in silico models indicate that the mechanism of action of vitamin A against COVID-19 is carried out via modulation of gene expression of key proteins involved in inflammatory reaction and reactive oxygen species production (such as MAPKs, IL10, ICAM1, or PRKCB) [56].

The intake of vitamin B_9_ (folate) significantly and inversely correlated with ∆I (Figure 1C), suggesting that poor consumption would be associated with faster SARS-CoV-2 expansion. In that context, it is remarkable that six (avocado, bananas, cereals, rice, pasta, and potatoes) of 27 foods located in the quadrant rotated component matrix associated with lower COVID-19 impact were rich in this vitamin. Folate is essential for the proper functioning of fundamental energy metabolism [57], T helpers, and NKs cell activity, as well as for adequate production of antibodies for B cells [58]. Accordingly, intake below the requirements of vitamin B_9_ is associated with imbalances and lower effectiveness of the immune response against infectious agents [59]. Additionally, it may play a role in protecting against COVID-19 and the severity caused by this disease: on the one hand, in silico models indicate that the structure of this micronutrient can physically interact and inhibit a convertase enzyme involved in the entry of SARS-CoV-2 into the host cell (called furin) [60]; on the other hand, vitamin B_9_ plays a key regulatory role in the homocysteine and one-carbon metabolic pathways [61]. Alterations in homocysteine levels and in folate-mediated one-carbon are implicated in pathology development (including Alzheimer or Type 2 Diabetes), and have been linked to increased cellular fragility and cell death rate by SARS-CoV-2 infection and long COVID suffering [61,62]. Moreover, recent evidence shows that the suboptimal nutritional status of folate and its relationship with COVID-19 could be a point of attention, especially in pregnant women [63]. Thus, although compliance with dietary recommendations of folate (or supplementation) in pregnancy is essential to promote normal newborn development [64], this need would result in an increase in the scenario of the COVID-19 pandemic. In this sense, ensuring optimal nutritional levels of folate during pregnancy would be a protective factor for pregnant women against COVID-19, but also for the newborn.

Zinc is another essential micronutrient in immunological terms with suboptimal consumption in the Spanish population. Furthermore, Figure 1C shows the strong inverse correlation between intake levels of zinc and higher COVID-19 ∆I in Spain. Although zinc did not correlate with any other epidemiological indicator, PCA results highlighted a pattern based on food consumption by territories associated with differential incidence/mortality of COVID-19 (Figure 3) and 37% of foods distinguished by the PCA were providing this essential mineral ACs (foods that provide content of this micronutrient equal to or greater than the average intake). These foods included cocoa powder, potatoes, beef, yogurts, whole wheat bread, turkey, and cheeses (fresh, semi-cured, and others). The dietary contribution of zinc is essential for the immune system, since it exerts important antioxidant [65,66] and anti-inflammatory functions that allow a more optimal immunological response [67]. Zinc deficiency is associated with immunological disorders, such as alterations in the phagocytic capacity of macrophages, the killing activity of NK cells, and in the T-cell-mediated antibody response [68]. Therefore, suboptimal consumption of this mineral would be associated with less capacity to properly respond to infections. In this way, patients critically affected by the Acute Respiratory Distress Syndrome caused by SARS-CoV-2 show a higher prevalence of low circulating levels of zinc [69]. The role of zinc as a prophylactic or complementary therapy for infection caused by SARS-CoV-2 is evaluated in several ongoing clinical trials [70]. For example, high oral doses of zinc (>15 mg/day) have been tested in hospitalized patients clinically diagnosed with COVID-19 resulting in improvement effects on COVID-19 symptomatology [71]. In addition, zinc therapy could shorten the duration of recovery of smell in patients with anosmia and/or hyposmia derived from COVID-19 [72]. These effects could have their mechanistic explanation in the ability of zinc to interact with SARS-CoV-2 RNA-polymerase and proteinases, essential elements for the infectivity process of the virus, according to the findings of studies of computer models [73].

Beyond these four micronutrients with estimated intakes below recommendations, intake levels of Vitamin B_12_ were also inversely associated with ∆I (Figure 1C). This vitamin is usually found in foods of animal origin. Although no suboptimal intake was observed in the Spanish population (Table 1), the vegan or vegetarian population must screen the nutritional status of this vitamin, since its deficiency is associated with a lower number of circulating lymphocytes and less responsiveness based on antibodies [45]. Thus, Vitamin B_12_ deficiency has been considered a modifiable risk factor for COVID-19 severity for population risk groups, such as diabetic and elderly population groups [74]. Moreover, B_12_ supplementation (in combination with vitamin D and Magnesium) has been associated with less need for oxygen support and/or intensive care in the elderly affected by the disease [75]. Regarding the rest of the analyzed micronutrients (vitamin C, vitamin B_6_, selenium, iron, and copper), intake estimation did not show any significant association between their intake and epidemiological indicators of COVID-19 (Figure 1C). However, this does not mean that they do not develop important roles in immune function or that their differential consumption is neutral for the incidence or severity of COVID-19. One plausible explanation of this could be that the intake level of vitamin C, vitamin B_6_, iron, and selenium would be close to the DRVs in most of the Spanish ACs, which could explain the lack of correlation when reaching the nutritional requirements for these micronutrients (Table 1). On the other hand, the estimated intake of copper showed the greatest variability when data from the different studies is compared (see Table 1). However, our results suggest a relevant role of the copper intake level, given the significant trend in the correlation tests with the incidence and I + M index parameters (Figure 1C), especially if the poor intake reported in this study (43.5% vs. DRV) and in other consulted sources (69.0%) (Table 1).

Given that our study was carried out on general population data, without discerning subgroups with COVID-19 risk factors (age, obesity, …), the results obtained in European, non-obese, healthy, physically active young people also highlight the importance of nutritional status on the incidence and severity of COVID-19 [76]. Thus, lower intakes of vitamin E, thiamine, and folate have been observed in subjects who contracted COVID-19 compared with the intakes of people who did not suffer from COVID-19. In addition, in the same study, it was observed that the daily intake of >10 g of nuts, rich in B vitamins, zinc, selenium, and copper (among other micronutrients) was associated with a lower risk of COVID-19 [76].

Finally, it should be mentioned that despite the similarity between the assessment of micronutrient intake in this study and the studies consulted, which have been carried out with different methodologies, one of the limitations of the present study may be the underestimation of intakes, for example, the potential punctual non-inclusion of foods that could be a source of any of the specific micronutrients in the assessment carried out. Besides, one of the potential factors that can influence the variability in the estimation of micronutrient intake could be related to the use of food composition data from the USDA database, as it included the info needed for this study. In this context, the current approach does not consider a possible variation in micronutrients associated with the soil content, which, in fact, has a strong influence on the final content in foods of plant origin. Specifically, the selenium and zinc content of plants can vary considerably depending on the soil richness of these micronutrients [77]. This could explain to some extent the discrepancy between the selenium intake collected in this study in comparison with others [78].

## 5. Conclusions

This study has considered the consumption needs of essential vitamins and minerals for the proper functioning of the immune system in Spain and in the Spanish regions. Low consumption of some micronutrients has been observed and associated with higher incidence and mortality from COVID-19, especially in specific zones of Spain. Despite the inherent technical limitations of ecological studies—including age distribution, disease status, socio-economic status, lifestyle factors, precautions taken against infection, stage of pandemic—data obtained on intake correspond with data collected from national or international studies already published. Considering the whole data set analyzed, consumption of vitamins D, A, B_9,_ and zinc would be suboptimal in the Spanish population, and this would be associated with a worse impact of COVID-19 in those areas with lower consumption of these four essential micronutrients. Specifically, Castilla-La Mancha, the Community of Navarre, La Rioja, and the Community of Madrid represented the Spanish areas with the highest COVID-19 incidence and mortality. Except for Navarre, the population of these CAs showed lower intake (below the national average) of vitamins D, A, B_9,_ and zinc. This landscape contrasted with the Spanish regions where the impact of the pandemic was lower (the Canary Islands, Galicia, Principality of Asturias, and the Valencian Community), which showed a significantly higher intake of these four micronutrients. Furthermore, correlation studies showed the association between vitamin D suboptimal consumption and the higher COVID-19 incidence/mortality in Spain, which fits with the results of epidemiological, interventional, clinical trials, and/or in vitro studies showing the benefits of vitamin D against COVID-19.

In conclusion, this report reveals the great relevance of the optimal consumption of essential micronutrients for the immune system and its relationship with the impact of COVID-19 in the different regions of Spain, highlighting the importance of vitamin D. Therefore, the information collected here can contribute to the development of food-based dietary guidelines, adapted to the gastronomy of each autonomous community and, especially, to the nutritional needs observed in each of them, to promote increased consumption of those missing micronutrients. Thus, these data can help the Spanish populations to achieve a more optimal nutritional status, promoting collective immune health, and preparing it to fight subsequent waves of COVID-19 and/or other infectious diseases.

## Figures and Tables

**Table 1:** Foremost micronutrients with a recognized contribution to the normal function of the immune system by EFSA; dietary reference values (DRV) for the adult population; Spanish median intake with the current approach and comparison with the intake reported from other studies.

	DRV for Adult Population	Intake Median (% DRV)	Published Intake Median (% DRV)	Accomplishment (%) [13]
**Vitamin D (µg/day)**	AI: 15	**2.46 (16.2%)**	**2.60 (17.3%)** [14]	**14.1**
**Vitamin A (µg/day)**	PRI: 650 (w)/750 (m)	**277 (39.9%)**	**477 (68.1%)** [15]	**77.2**
Vitamin C (mg/day)	PRI: 95 (w)/110 (m)	95.0 (91.9%)	71.3 (69.6%) [15]	110
Vitamin B_6_ (mg/day)	AI: 1.6 (w)/1.7 (m)	1.40 (83.2%)	1.44 (87.3%) [16]	112
**Vitamin B_9_ (µg/day)**	PRI: 330	**238 (71.1%)**	**160 (48.4%)** [17]	**74.9**
Vitamin B_12_ (µg/day)	PRI: 4	5.23 (132%)	4.20 (105%) [17]	128
**Zinc (mg/day)**	PRI *: 10.1 (w)/10.9 (m)	**4.42 (38.6%)**	**7.70 (67.0%)** [14]	**81.2**
Iron (mg/day)	PRI: 11	7.96 (72.8%)	10.5 (95.7%) [16]	111
Copper (mg/day)	PRI: 1.3 (w)/1.6 (m)	0.62 (43.5%)	1.00 (69.0%) [18]	115
Selenium (µg/day)	AI: 70	92.3 (131%)	72.0 (103%) [15]	108

Dietary Reference Intakes (DRV) were collected from DRV Finder Tool [19] supported by the European Food Safety Authority (EFSA). Zinc PRI * is expressed as the global average of all the values co-dependent on the level of phytate intake (300, 600, 900 and 1200 mg/day). The intake and the published intake are expressed as median and the percentage of this median vs. DRV. Accomplishment is the percentage of the observed intake referred to the established requirements (as 100% of PRI value) collected from [13]. In bold are highlighted those micronutrients with suboptimal intake values in the Spanish population according to both, calculated and consulted data. Abbreviations: AI (Average Intake); PRI (Population Reference Intake); (w) and (m) mean recommendations for women and males, respectively.

**Table 2:** COVID-19 epidemiological indicators per regions during the exponential phase of the second wave (Autonomous Communities, ACs) of Spain ^1^.

ACs	Prevalence (P)	Incidence -45 (I_-45_)	Incidence (I)	Δ Incidence (ΔI)	Mortality (M)	I + M (Z-score)
Canary Islands	3.8	445.9	768.4	1.7	12.4	−2.5
Galicia	4.5	617.1	1063.6	1.7	31.8	−1.8
Principality of Asturias	6.1	367.4	922.2	2.5	38.2	−1.8
Valencian Community	5.7	584.6	1075.8	1.8	34.4	−1.8
Andalusia	7.1	450.5	1263.3	2.8	27.0	−1.8
Balearic Islands	6.3	881.7	1468.2	1.7	30.6	−1.5
Region of Murcia	6.1	630.5	1958.2	3.1	18.9	−1.3
Cantabria	6.3	868.9	1462.8	1.7	42.7	−1.3
Extremadura	8.0	585.0	1505.8	2.6	63.3	−0.9
Catalonia	11.6	1561.4	2502.0	1.6	77.7	0.2
Basque Country	8.2	1578.6	2610.2	1.7	92.0	0.6
Castilla y León	12.6	1372.0	2916.8	2.1	143.6	1.9
Aragon	11.7	2191.6	3659.7	1.7	118.7	2.0
Castilla-La Mancha	16.1	1367.9	2821.0	2.1	164.2	2.2
CC of Navarre	14.3	1770.4	4281.3	2.4	102.1	2.2
La Rioja	8.2	2014.2	3566.9	1.8	139.5	2.3
Community of Madrid	18.6	2283.9	4393.5	1.9	153.4	3.3

^1^ The ACs list is arranged according to the I + M value, in ascending order. The dashed lines divide the table according to quartile (Q) of I + M index: Q1 (top), Q2 + Q3 (in the center), and Q4 (bottom) Abbreviations: ACs (Autonomous Community); P (Prevalence, expressed as % of the population with IgG anti-SARS-CoV-2); I_-45_ (Incidence, expressed as the accumulated number of cases per 100 k people until 9 September 2020); I (Incidence, expressed as the accumulated number of cases per 100 k people until 23 October 2020); ΔI (Incidence increase is expressed as I/I_-45_); M (Mortality rate, as the number of dead people due to COVID-19 per 1 M people), and I + M (is expressed as the addition of I + M Z-scores).

## Supplementary Materials

The following supporting information can be downloaded at: https://www.mdpi.com/article/10.3390/nu14112254/s1, Table S1: Intake of 10 analyzed micronutrients by Autonomous Communities of Spain. Table S2: Complete list of food items selected for the preparation of the Principal Component Analysis and Rotated Component Matrix value for Principal Components 1 and 2.

## References

[1] World Health Organization WHO Coronavirus Disease (COVID-19) Dashboard. https://covid19.who.int/.

[2] Wang H., Paulson K.R., Pease S.A., Watson S., Comfort H., Zheng P., Aravkin A.Y., Bisignano C., Barber R.M., Alam T. (2022). Estimating excess mortality due to the COVID-19 pandemic: A systematic analysis of COVID-19-related mortality, 2020–2021. Lancet.

[3] Gombart A.F., Pierre A., Maggini S. (2020). A Review of Micronutrients and the Immune System–Working in Harmony to Reduce the Risk of Infection. Nutrients.

[4] Hotamisligil G.S. (2017). Inflammation, metaflammation and immunometabolic disorders. Nature.

[5] EFSA Panel on Dietetic Products Nutrition and Allergies (NDA) (2015). Scientific Opinion on the substantiation of a health claim related to vitamin D and contribution to the normal function of the immune system pursuant to Article 14 of Regulation (EC) No 1924/2006. EFSA J..

[6] EFSA Panel on Dietetic Products Nutrition and Allergies (NDA) (2010). Scientific Opinion on the substantiation of health claims related to vitamin D and normal function of the immune system and inflammatory response (ID 154, 159), maintenance of normal muscle function (ID 155) and maintenance of normal cardiovascular functi. EFSA J..

[7] EFSA Panel on Dietetic Products Nutrition and Allergies (NDA) (2009). Scientific Opinion on the substantiation of health claims related to vitamin B6 and protein and glycogen metabolism (ID 65, 70, 71), function of the nervous system (ID 66), red blood cell formation (ID 67, 72, 186), function of the immune system (ID 68). EFSA J..

[8] EFSA Panel on Dietetic Products Nutrition and Allergies (NDA) (2009). Scientific Opinion on the substantiation of health claims related to zinc and function of the immune system (ID 291, 1757), DNA synthesis and cell division (ID 292, 1759), protection of DNA, proteins and lipids from oxidative damage (ID 294, 1758), mainte. EFSA J..

[9] EFSA Panel on Dietetic Products Nutrition and Allergies (NDA) (2009). Scientific Opinion on the substantiation of health claims related to iron and formation of red blood cells and haemoglobin (ID 249, ID 1589), oxygen transport (ID 250, ID 254, ID 256), energy-yielding metabolism (ID 251, ID 1589), function of the immune s. EFSA J..

[10] EFSA Panel on Dietetic Products Nutrition and Allergies (NDA) (2009). Scientific Opinion on the substantiation of health claims related to copper and protection of DNA, proteins and lipids from oxidative damage (ID 263, 1726), function of the immune system (ID 264), maintenance of connective tissues (ID 265, 271, 1722), ene. EFSA J..

[11] EFSA Panel on Dietetic Products Nutrition and Allergies (NDA) (2011). Scientific Opinion on the substantiation of health claims related to copper and reduction of tiredness and fatigue (ID 272), maintenance of the normal function of the nervous system (ID 1723), maintenance of the normal function of the immune system (ID 17. EFSA J..

[12] EFSA Panel on Dietetic Products Nutrition and Allergies (NDA) (2009). Scientific Opinion on the substantiation of health claims related to selenium and protection of DNA, proteins and lipids from oxidative damage (ID 277, 283, 286, 1289, 1290, 1291, 1293, 1751), function of the immune system (ID 278), thyroid function (ID 2. EFSA J..

[13] Galmés S., Serra F., Palou A. (2020). Current State of Evidence: Influence of Nutritional and Nutrigenetic Factors on Immunity in the COVID-19 Pandemic Framework. Nutrients.

[14] Olza J., Aranceta-Bartrina J., González-Gross M., Ortega R., Serra-Majem L., Varela-Moreiras G., Gil Á. (2017). Reported Dietary Intake, Disparity between the Reported Consumption and the Level Needed for Adequacy and Food Sources of Calcium, Phosphorus, Magnesium and Vitamin D in the Spanish Population: Findings from the ANIBES Study. Nutrients.

[15] Olza J., Aranceta-Bartrina J., González-Gross M., Ortega R., Serra-Majem L., Varela-Moreiras G., Gil Á. (2017). Reported Dietary Intake and Food Sources of Zinc, Selenium, and Vitamins A, E and C in the Spanish Population: Findings from the ANIBES Study. Nutrients.

[16] Mielgo-Ayuso J., Aparicio-Ugarriza R., Olza J., Aranceta-Bartrina J., Gil Á., Ortega R.M., Serra-Majem L., Varela-Moreiras G., González-Gross M. (2018). Dietary intake and food sources of niacin, riboflavin, thiamin and vitamin B6 in a representative sample of the spanish population. The anthropometry, intake, and energy balance in Spain (ANIBES) study. Nutrients.

[17] Partearroyo T., De Lourdes Samaniego-Vaesken M., Ruiz E., Olza J., Aranceta-Bartrina J., Gil Á., González-Gross M., Ortega R.M., Serra-Majem L., Varela-Moreiras G. (2017). Dietary sources and intakes of folates and Vitamin B12 in the Spanish population: Findings from the ANIBES study. PLoS ONE.

[18] Goñi I., Hernández-Galiot A. (2019). Intake of Nutrient and Non-Nutrient Dietary Antioxidants. Contribution of Macromolecular Antioxidant Polyphenols in an Elderly Mediterranean Population. Nutrients.

[19] European Food Safety Authority (EFSA) DRV Finder. https://www.efsa.europa.eu/en/interactive-pages/drvs.

[20] Annual Data of the Panel of Food Consumption in Households. https://www.mapa.gob.es/es/alimentacion/temas/consumo-tendencias/panel-de-consumo-alimentario/series-anuales/default.aspx.

[21] Update no 235 of the Coronavirus Disease (COVID-19). Health Ministry of the Spanish Government. https://www.sanidad.gob.es/profesionales/saludPublica/ccayes/alertasActual/nCov/documentos/Actualizacion_203_COVID-19.pdf..

[22] Update no 203 of the Coronavirus Disease (COVID-19). Health Ministry of the Spanish Government. https://www.sanidad.gob.es/profesionales/saludPublica/ccayes/alertasActual/nCov/documentos/Actualizacion_235_COVID-19.pdf..

[23] (2020). Estudio ENE-COVID: Cuarta Ronda Estudio Nacional de Sero-Epidemiología de la Infección por SARS-COV-2 en España. https://www.sanidad.gob.es/gabinetePrensa/notaPrensa/pdf/15.12151220163348113.pdf..

[24] STHDA—Home. http://www.sthda.com/english/.

[25] European Food Safety Authority (EFSA) (2017). Dietary Reference Values for nutrients Summary report. EFSA Support. Publ..

[26] European Food Safety Authority (EFSA) (2013). Scientific Opinion on Dietary Reference Values for energy. EFSA J..

[27] Gamero-de-Luna E.J., Gamero-Estévez E. (2021). Mutaciones, variantes y cepas de SARS-CoV-2. Med. Fam. Semer..

[28] Faurschou A., Beyer D.M., Schmedes A., Bogh M.K., Philipsen P.A., Wulf H.C. (2012). The relation between sunscreen layer thickness and vitamin D production after ultraviolet B exposure: A randomized clinical trial. Br. J. Dermatol..

[29] Fayet-Moore F., Brock K.E., Wright J., Ridges L., Small P., Seibel M.J., Conigrave A.D., Mason R.S. (2019). Determinants of vitamin D status of healthy office workers in Sydney, Australia. J. Steroid Biochem. Mol. Biol..

[30] EFSA Panel on Dietetic Products, Nutrition and Allergies (2016). Dietary reference values for vitamin D. EFSA J..

[31] Carr A.C., Gombart A.F. (2022). Multi-Level Immune Support by Vitamins C and D during the SARS-CoV-2 Pandemic. Nutrients.

[32] Ilie P.C., Stefanescu S., Smith L. (2020). The role of vitamin D in the prevention of coronavirus disease 2019 infection and mortality. Aging Clin. Exp. Res..

[33] Ruan Q., Yang K., Wang W., Jiang L., Song J. (2020). Clinical predictors of mortality due to COVID-19 based on an analysis of data of 150 patients from Wuhan, China. Intensive Care Med..

[34] Alipio M. (2020). Vitamin D Supplementation Could Possibly Improve Clinical Outcomes of Patients Infected with Coronavirus-2019 (COVID-2019). SSRN Electron. J..

[35] Daneshkhah A., Eshein A., Subramanian H., Roy H.K., Backman V. (2020). The Role of Vitamin D in Suppressing Cytokine Storm in COVID-19 Patients and Associated Mortality. medRxiv.

[36] Grant W.B., Al Anouti F., Boucher B.J., Dursun E., Gezen-Ak D., Jude E.B., Karonova T., Pludowski P. (2022). A Narrative Review of the Evidence for Variations in Serum 25-Hydroxyvitamin D Concentration Thresholds for Optimal Health. Nutrients.

[37] Karonova T.L., Chernikova A.T., Golovatyuk K.A., Bykova E.S., Grant W.B., Kalinina O.V., Grineva E.N., Shlyakhto E.V. (2022). Vitamin D Intake May Reduce SARS-CoV-2 Infection Morbidity in Health Care Workers. Nutrients.

[38] Benedik E. (2022). Sources of vitamin D for humans. Int. J. Vitam. Nutr. Res..

[39] Sassi F., Tamone C., D’Amelio P. (2018). Vitamin D: Nutrient, Hormone, and Immunomodulator. Nutrients.

[40] Bertoldi G., Gianesello L., Calò L.A. (2020). ACE2, Rho kinase inhibition and the potential role of vitamin D against COVID-19. Aliment. Pharmacol. Ther..

[41] Grant W.B., Lahore H., McDonnell S.L., Baggerly C.A., French C.B., Aliano J.L., Bhattoa H.P. (2020). Evidence that vitamin d supplementation could reduce risk of influenza and COVID-19 infections and deaths. Nutrients.

[42] Huang C., Wang Y., Li X., Ren L., Zhao J., Hu Y., Zhang L., Fan G., Xu J., Gu X. (2020). Clinical features of patients infected with 2019 novel coronavirus in Wuhan, China. Lancet.

[43] Bonet M.L., Ribot J., Galmés S., Serra F., Palou A. (2020). Carotenoids and carotenoid conversion products in adipose tissue biology and obesity: Pre-clinical and human studies. Biochim. Biophys. Acta-Mol. Cell Biol. Lipids.

[44] Kańtoch M., Litwińska B., Szkoda M., Siennicka J. (2002). Importance of vitamin A deficiency in pathology and immunology of viral infections. Rocz. Panstw. Zakl. Hig..

[45] Maggini S., Wintergerst E.S., Beveridge S., Hornig D.H. (2007). Selected vitamins and trace elements support immune function by strengthening epithelial barriers and cellular and humoral immune responses. Br. J. Nutr..

[46] Chew B.P. (1993). Role of Carotenoids in the Immune Response. J. Dairy Sci..

[47] Toti E., Oliver Chen C.Y., Palmery M., Valencia D.V., Peluso I. (2018). Non-provitamin A and provitamin A carotenoids as immunomodulators: Recommended dietary allowance, therapeutic index, or personalized nutrition?. Oxid. Med. Cell. Longev..

[48] Sarohan A.R. (2020). COVID-19: Endogenous Retinoic Acid Theory and Retinoic Acid Depletion Syndrome. Med. Hypotheses.

[49] Al-Saleh I., Alrushud N., Alnuwaysir H., Elkhatib R., Shoukri M., Aldayel F., Bakheet R., Almozaini M. (2022). Essential metals, vitamins and antioxidant enzyme activities in COVID-19 patients and their potential associations with the disease severity. Biometals.

[50] Semba R.D. (1999). Vitamin A and immunity to viral, bacterial and protozoan infections. Proc. Nutr. Soc..

[51] Villamor E., Mbise R., Spiegelman D., Hertzmark E., Fataki M., Peterson K.E., Ndossi G., Fawzi W.W. (2002). Vitamin A Supplements Ameliorate the Adverse Effect of HIV-1, Malaria, and Diarrheal Infections on Child Growth. Pediatrics.

[52] Stockman L.J., Bellamy R., Garner P. (2006). SARS: Systematic Review of Treatment Effects. PLoS Med..

[53] Trasino S.E. (2020). A role for retinoids in the treatment of COVID-19?. Clin. Exp. Pharmacol. Physiol..

[54] Chen K.-H., Wang S.-F., Wang S.-Y., Yang Y.-P., Wang M.-L., Chiou S.-H., Chang Y.-L. (2020). Pharmacological development of the potential adjuvant therapeutic agents against coronavirus disease 2019. J. Chin. Med. Assoc..

[55] Midha I.K., Kumar N., Kumar A., Madan T. (2020). Mega doses of retinol: A possible immunomodulation in Covid-19 illness in resource-limited settings. Rev. Med. Virol..

[56] Li R., Wu K., Li Y., Liang X., Fai Tse W.K., Yang L., Lai K.P. (2020). Revealing the targets and mechanisms of vitamin A in the treatment of COVID-19. Aging.

[57] Ducker G.S., Rabinowitz J.D. (2017). One-Carbon Metabolism in Health and Disease. Cell Metab..

[58] Saeed F., Nadeem M., Ahmed R.S., Tahir Nadeem M., Arshad M.S., Ullah A. (2016). Studying the impact of nutritional immunology underlying the modulation of immune responses by nutritional compounds—A review. Food Agric. Immunol..

[59] Troen A.M., Mitchell B., Sorensen B., Wener M.H., Johnston A., Wood B., Selhub J., McTiernan A., Yasui Y., Oral E. (2006). Unmetabolized Folic Acid in Plasma Is Associated with Reduced Natural Killer Cell Cytotoxicity among Postmenopausal Women. J. Nutr..

[60] Sheybani Z., Dokoohaki M.H., Negahdaripour M., Dehdashti M., Zolghadr H., Moghadami M., Masoom Masoompour S., Zolghadr A.R. (2020). The role of folic acid in the management of respiratory disease caused by COVID-19. ChemRxiv.

[61] Singh Y., Gupta G., Kazmi I., Al-Abbasi F.A., Negi P., Chellappan D., Dua K. (2020). SARS CoV-2 aggravates cellular metabolism mediated complications in COVID-19 infection. Dermatol. Ther..

[62] Hayden M.R., Tyagi S.C. (2021). Impaired Folate-Mediated One-Carbon Metabolism in Type 2 Diabetes, Late-Onset Alzheimer’s Disease and Long COVID. Medicina.

[63] Acosta-Elias J., Espinosa-Tanguma R. (2020). The Folate Concentration and/or Folic Acid Metabolites in Plasma as Factor for COVID-19 Infection. Front. Pharmacol..

[64] Uta M., Neamtu R., Bernad E., Mocanu A.G., Gluhovschi A., Popescu A., Dahma G., Dumitru C., Stelea L., Citu C. (2022). The Influence of Nutritional Supplementation for Iron Deficiency Anemia on Pregnancies Associated with SARS-CoV-2 Infection. Nutrients.

[65] Topilski I., Flaishon L., Naveh Y., Harmelin A., Levo Y., Shachar I. (2004). The anti-inflammatory effects of 1,25-dihydroxyvitamin D3 on Th2 cellsin vivo are due in part to the control of integrin-mediated T lymphocyte homing. Eur. J. Immunol..

[66] Maggini S. (2008). Feeding the immune system: The role of micronutrients in restoring resistance to infections. CAB Rev. Perspect. Agric. Vet. Sci. Nutr. Nat. Resour..

[67] de Almeida Brasiel P.G. (2020). The key role of zinc in elderly immunity: A possible approach in the COVID-19 crisis. Clin. Nutr. ESPEN.

[68] Tuerk M.J., Fazel N. (2009). Zinc deficiency. Curr. Opin. Gastroenterol..

[69] Gonçalves T.J.M., Gonçalves S.E.A.B., Guarnieri A., Risegato R.C., Guimarães M.P., de Freitas D.C., Razuk-Filho A., Junior P.B.B., Parrillo E.F. (2020). Association between Low Zinc Levels and Severity of Acute Respiratory Distress Syndrome by New Coronavirus (SARS-CoV-2). Nutr. Clin. Pract..

[70] Joachimiak M.P. (2021). Zinc against COVID-19? Symptom surveillance and deficiency risk groups. PLoS Negl. Trop. Dis..

[71] Finzi E. (2020). Treatment of SARS-CoV-2 with high dose oral zinc salts: A report on four patients. Int. J. Infect. Dis..

[72] Abdelmaksoud A.A., Ghweil A.A., Hassan M.H., Rashad A., Khodeary A., Aref Z.F., Sayed M.A.A., Elsamman M.K., Bazeed S.E.S. (2021). Olfactory Disturbances as Presenting Manifestation Among Egyptian Patients with COVID-19: Possible Role of Zinc. Biol. Trace Elem. Res..

[73] Pormohammad A., Monych N., Turner R. (2020). Zinc and SARS-CoV-2: A molecular modeling study of Zn interactions with RNA-dependent RNA-polymerase and 3C-like proteinase enzymes. Int. J. Mol. Med..

[74] Wee A.K.H. (2020). COVID-19′s toll on the elderly and those with diabetes mellitus—Is vitamin B12 deficiency an accomplice?. Med. Hypotheses.

[75] Tan C.W., Ho L.P., Kalimuddin S., Cherng B.P.Z., Teh Y.E., Thien S.Y., Wong H.M., Tern P.J.W., Chandran M., Chay J.W.M. (2020). Cohort study to evaluate the effect of vitamin D, magnesium, and vitamin B12 in combination on progression to severe outcomes in older patients with coronavirus (COVID-19). Nutrition.

[76] Jagielski P., Łuszczki E., Wnęk D., Micek A., Bolesławska I., Piórecka B., Kawalec P. (2022). Associations of Nutritional Behavior and Gut Microbiota with the Risk of COVID-19 in Healthy Young Adults in Poland. Nutrients.

[77] Gupta U.C., Gupta S.C. (2000). Selenium in soils and crops, its deficiencies in livestock and humans: Implications for management. Commun. Soil Sci. Plant Anal..

[78] Jones G.D., Droz B., Greve P., Gottschalk P., Poffet D., McGrath S.P., Seneviratne S.I., Smith P., Winkel L.H.E. (2017). Selenium deficiency risk predicted to increase under future climate change. Proc. Natl. Acad. Sci. USA.

