# Supplementary material for: Suboptimal Consumption of Relevant Immune System Micronutrients Is Associated with a Worse Impact of COVID-19 in Spanish Populations
Source: Nutrients. 2022 May 27;14(11):2254. doi: 10.3390/nu14112254 (PMC9183133; doi:10.3390/nu14112254)
Supplement: Supplementary file 1 [file nutrients-14-02254-s001.zip › nutrients-1724181-supplementary.pdf]

**Supplementary Table S1.** Estimated intake of 10 analyzed micronutrients by Autonomous Communities (ACs) of Spain. The data are expressed as intake values (μg or mg) per day per person in each ACs and the corresponding percentage that it supposes with respect to the nutritional requirements for adults (in parentheses).

| | **Vit. D (μg/day)** | **Vit. A (μg/day)** | Vit. C (mg/day) | Vit. $B_6$ (mg/day) | **Vit. $B_9$ (μg/day)** | Vit. $B_{12}$ (μg/day) | **Zinc (mg/day)** | Iron (mg/day) | Copper (mg/day) | Selenium (μg/day) |
|---|---|---|---|---|---|---|---|---|---|---|
| Andalusia | 2.23 (14.9%) | 282 (40.3%) | 81.9 (80%) | 1.30 (78.7%) | 211 (64.0%) | 4.42 (111%) | 3.83 (33.3%) | 7.21 (65.5%) | 0.59 (41.0%) | 84.3 (120%) |
| Aragon | 2.35 (15.6%) | 270 (38.6%) | 94.9 (92.6%) | 1.29 (78.3%) | 218 (66.2%) | 5.33 (133%) | 4.47 (38.9%) | 7.19 (65.3%) | 0.58 (39.7%) | 88.2 (126%) |
| Principality of Asturias | 2.82 (18.8%) | 277 (39.5%) | 105 (102%) | 1.37 (82.8%) | 224 (68.0%) | 6.08 (152%) | 4.62 (40.1%) | 8.46 (76.9%) | 0.67 (46.3%) | 99.2 (142%) |
| Balearic Islands | 2.62 (17.4%) | 358 (51.1%) | 96.6 (94.3%) | 1.58 (95.8%) | 279 (84.5%) | 5.43 (136%) | 4.96 (43.1%) | 8.69 (79.0%) | 0.68 (47.1%) | 95.0 (136%) |
| Canary Islands | 2.62 (17.5%) | 306 (43.7%) | 82.1 (80.1%) | 1.45 (88.1%) | 238 (72.0%) | 4.74 (119%) | 4.19 (36.4%) | 7.96 (72.4%) | 0.62 (42.5%) | 81.5 (116%) |
| Cantabria | 2.49 (16.6%) | 280 (40.0%) | 97.2 (94.9%) | 1.43 (86.5%) | 254 (76.9%) | 5.27 (132%) | 4.71 (41.0%) | 8.75 (79.6%) | 0.71 (49.3%) | 96.3 (138%) |
| Castilla-La Mancha | 2.20 (14.7%) | 233 (33.3%) | 84.5 (82.4%) | 1.26 (76.2%) | 207 (62.8%) | 4.71 (118%) | 4.14 (36.0%) | 7.18 (65.3%) | 0.6 (41.1%) | 87.3 (125%) |
| Castilla y León | 2.61 (17.4%) | 296 (42.2%) | 108 (105%) | 1.43 (86.4%) | 242 (73.3%) | 6.15 (154%) | 5.00 (43.5%) | 8.92 (81.1%) | 0.67 (45.9%) | 105 (150%) |
| Catalonia | 2.46 (16.4%) | 301 (43.0%) | 102 (100%) | 1.45 (87.7%) | 257 (77.8%) | 5.53 (138%) | 4.61 (40.1%) | 8.02 (72.9%) | 0.62 (42.8%) | 92.9 (133%) |
| Valencian Community | 2.51 (16.7%) | 291 (41.5%) | 85.8 (83.7%) | 1.40 (84.6%) | 244 (73.8%) | 5.23 (131%) | 4.52 (39.3%) | 7.84 (71.2%) | 0.61 (41.8%) | 92.3 (132%) |
| Extremadura | 2.11 (14.1%) | 273 (39.1%) | 86.8 (84.7%) | 1.19 (71.9%) | 188 (57.1%) | 4.18 (105%) | 3.64 (31.7%) | 6.66 (60.5%) | 0.59 (40.7%) | 79.6 (114%) |
| Galicia | 2.48 (16.6%) | 271 (38.8%) | 109 (106%) | 1.54 (93.4%) | 251 (76.0%) | 6.69 (167%) | 5.17 (45.0%) | 9.57 (87.0%) | 0.74 (50.8%) | 108 (154%) |
| Community of Madrid | 2.37 (15.8%) | 277 (39.5%) | 94.6 (92.3%) | 1.35 (81.8%) | 222 (67.1%) | 5.14 (128%) | 4.32 (38.0%) | 7.27 (66.1%) | 0.56 (38.6%) | 83.9 (120%) |
| Region of Murcia | 2.45 (16.3%) | 256 (36.6%) | 81.7 (79.7%) | 1.31 (79.2%) | 232 (70.4%) | 4.70 (117%) | 3.97 (34.5%) | 7.57 (68.8%) | 0.59 (40.9%) | 83.8 (120%) |
| Chartered Community of Navarre | 2.24 (15.0%) | 264 (37.8%) | 106 (104%) | 1.46 (88.4%) | 268 (81.2%) | 5.05 (126%) | 4.55 (39.5%) | 8.72 (79.3%) | 0.67 (45.9%) | 94.9 (136%) |
| Basque Country | 2.52 (16.8%) | 294 (42.0%) | 108 (106%) | 1.43 (86.8%) | 253 (76.7%) | 5.79 (145%) | 4.77 (41.5%) | 9.00 (81.8%) | 0.71 (48.8%) | 98.7 (141%) |
| La Rioja | 2.26 (15.1%) | 221 (31.5%) | 76.4 (74.6%) | 1.12 (68.1%) | 202 (61.1%) | 5.00 (125%) | 4.05 (35.2%) | 7.18 (65.3%) | 0.53 (36.4%) | 86.1 (123%. |

**Supplementary Table S2.** Complete list of food items selected for the preparation of the Principal Component Analysis and Rotated Component Matrix value for Principal Components 1 and 2.

| Item | PC1 | PC2 | Item | PC1 | PC2 |
|---|---|---|---|---|---|
| Negative PC1 / Positive PC2 quadrant | | | Positives PC1 and PC2 quadrant | | |
| Juices/Nectar | -0.87 | 0.07 | Onions | 0.03 | 0.47 |
| Tuna | -0.86 | 0.17 | Peppers | 0.10 | 0.44 |
| Cheese (other) | -0.73 | 0.50 | Olive oils | 0.18 | 0.77 |
| Avocado | -0.67 | 0.30 | Lentils | 0.26 | 0.49 |
| Mackerel | -0.66 | 0.31 | Kiwi | 0.29 | 0.86 |
| Ham | -0.65 | 0.46 | Beans | 0.36 | 0.15 |
| Butter | -0.63 | 0.52 | Semi Skimmed Milk | 0.36 | 0.05 |
| Semi-cured Cheese | -0.62 | 0.04 | Squid/Octopus | 0.38 | 0.42 |
| Cereals | -0.53 | 0.31 | Whole Milk | 0.38 | 0.45 |
| Margarine | -0.50 | 0.01 | Chocolate (others) | 0.38 | 0.46 |
| Flours/Semolina | -0.49 | 0.37 | Skimmed Milk | 0.40 | 0.76 |
| Turkey | -0.47 | 0.01 | Garlic | 0.42 | 0.03 |
| Spices | -0.38 | 0.18 | Eggs | 0.45 | 0.60 |
| Cream | -0.33 | 0.01 | Bread | 0.46 | 0.72 |
| Yogurts | -0.32 | 0.67 | Cockles/Clams | 0.48 | 0.32 |
| Carrots | -0.30 | 0.49 | Turkey | 0.49 | 0.07 |
| Bananas | -0.30 | 0.70 | Oranges | 0.51 | 0.75 |
| Pasta | -0.28 | 0.11 | Praws | 0.52 | 0.06 |
| Beef | -0.24 | 0.48 | Lettuce/Endive | 0.57 | 0.07 |
| Potatoes | -0.20 | 0.55 | Mussels | 0.59 | 0.22 |
| Fresh Cheese | -0.20 | 0.41 | Veal | 0.63 | 0.65 |
| Skimmed yogurt | -0.18 | 0.70 | Pineapple | 0.67 | 0.18 |
| Canned tuna | -0.16 | 0.61 | Strawberries | 0.72 | 0.46 |
| Lemons | -0.12 | 0.03 | Tangerines | 0.76 | 0.33 |
| Cocoa Powder | -0.02 | 0.47 | Bass | 0.79 | 0.12 |
| | | | Hake | 0.82 | 0.39 |
| Negatives PC1 and PC2 quadrant | | | Positive PC1 / negative PC2 quadrant | | |
| Rice | -0.58 | -0.06 | Chickpea | 0.02 | -0.08 |
| Wholemeal bread | -0.39 | -0.05 | Mushrooms | 0.07 | -0.29 |
| Foie Gras and Patés | -0.34 | -0.66 | Calcium fortified milk | 0.14 | -0.42 |
| Watermelon | -0.14 | -0.70 | Artichoke | 0.16 | -0.55 |
| Tomatoes | -0.08 | -0.57 | Melon | 0.18 | -0.40 |
| | | | Cured Ham | 0.41 | -0.35 |
| | | | Pork Meat | 0.64 | -0.02 |
| | | | Salmon | 0.66 | -0.22 |
| | | | Chicken meat | 0.70 | -0.37 |
| | | | Lamb meat | 0.76 | -0.29 |

[1] The foods appear ordered according to their location in the PCA quadrants (see Figure 3B).
